# Early intervention to protect the mother-infant relationship following postnatal depression: study protocol for a randomised controlled trial

**DOI:** 10.1186/1745-6215-15-385

**Published:** 2014-10-03

**Authors:** Jeannette Milgrom, Charlene Holt

**Affiliations:** Parent-Infant Research Institute, Austin Health, 300 Waterdale Rd, Heidelberg Heights, VIC 3081 Australia; Melbourne School of Psychological Sciences, University of Melbourne, Grattan St, Parkville, VIC 3010 Australia

**Keywords:** Postnatal depression, Mother-infant difficulties, Randomised controlled trial

## Abstract

**Background:**

At least 13% of mothers experience depression in the first postnatal year, with accompanying feelings of despair and a range of debilitating symptoms. Serious sequelae include disturbances in the mother-infant relationship and poor long-term cognitive and behavioural outcomes for the child. Surprisingly, treatment of maternal symptoms of postnatal depression does not improve the mother-infant relationship for a majority of women. Targeted interventions to improve the mother-infant relationship following postnatal depression are scarce and, of those that exist, the majority are not evaluated in randomised controlled trials. This study aims to evaluate a brief targeted mother-infant intervention, to follow cognitive behavioural therapy treatment of postnatal depression, which has the potential to improve developmental outcomes of children of depressed mothers.

**Methods/Design:**

The proposed study is a two-arm randomised controlled trial with follow-up to 6 months. One hundred participants will be recruited via referrals from health professionals including maternal and child health nurses and general practitioners, as well as self-referrals from women who have seen promotional materials for the study. Women who meet inclusion criteria (infant aged <12 months, women 18+ years of age) will complete the Structured Clinical Interview for the Diagnostic and Statistical Manual of Mental Disorders-IV-TR Axis I Disorders. Those with a clinical diagnosis of current major or minor depressive disorder and who do not meet exclusion criteria (that is, currently receiving treatment for depression, significant difficulty with English, medium to high suicide risk, current self-harm, current substance abuse, current post-traumatic stress disorder, current manic/hypomanic episode or psychotic symptoms) will be randomised to receive either a 4-session mother-infant intervention (HUGS: Happiness Understanding Giving and Sharing) or a 4-session attention placebo playgroup (Playtime) following a 12-session postnatal depression group treatment programme. Primary outcome measures are the Parenting Stress Index (self-report measure) and the Parent-child Early Relational Assessment (observational measure coded by a blinded observer). Measurements are taken at baseline, after the postnatal depression programme, post-HUGS/Playtime, and at 6 months post-HUGS/Playtime.

**Discussion:**

This research addresses the need for specific treatment for mother-infant interactional difficulties following postnatal depression. There is a need to investigate interventions in randomised trials to prevent detrimental effects on child development and make available evidence-based treatments.

**Trial registration:**

Australia and New Zealand Clinical Trials Register: ACTRN12612001110875. Date Registered: 17 October 2012.

**Electronic supplementary material:**

The online version of this article (doi:10.1186/1745-6215-15-385) contains supplementary material, which is available to authorized users.

## Background

Around 7.1% of new mothers have a major depressive episode during the first 3 months after delivery with an additional 12.1% of women suffering minor depression[1]. Postnatal depression (PND) is accompanied by a range of disturbing symptoms which can have a profound effect on the mother. Symptoms can include depressed mood, loss of interest, weight loss or gain, sleep disturbance, lack of energy, feeling agitated or slowed down, feelings of worthlessness or guilt, loss of concentration, and thoughts of death or suicide[2]. High anxiety is often co-morbid[3]. Symptoms of PND take on a particular significance due to the presence of an infant.

### Interactional difficulties accompanying postnatal depression

Current research strongly suggests that PND interferes with the behavioural and emotional exchanges between mother and infant[4]. Depressed mothers gaze less at their infants, rock their infants less, are less active and decisive, have less well-timed responsiveness, demonstrate lower levels of warm acceptance, are emotionally flat, and often disengaged[5–8]. Our earlier work demonstrated that infant development is powerfully shaped by the quality of the early mother-infant interaction following PND[9, 10]. These interactions are complicated, involving reciprocal, inter-dependent effects between child and mother. Brazelton and colleagues[11], Tronick and Weinberg[12], and Stern[13] describe the critical elements of a successful interaction, which includes an emotionally attuned and responsive mother. Emotional unavailability following depression may result in an escalating cycle of dysfunctional behaviours in both mother and child (for instance, maternal flat affect leading to gaze aversion in the infant leading to feelings of rejection in the mother and withdrawal).

### Importance of early mother-infant relationships

There is a growing awareness of the importance of early experiences in shaping infant brain development. Due to the plasticity of the brain at this early developmental stage, stressful experiences, including interactions with an un-attuned caregiver, may evoke permanent changes in brain organization[14–16]. The National Forum on Early Childhood Program Evaluation[17] concluded that infants of women with PND may experience “lasting effects on their brain architecture and persistent disruptions of their stress response systems” (page 3). Cognitive, emotional and social capabilities are all inextricably linked in brain development[18]. During the first 3 years of life, brain development is at its fastest and the brain is at its most malleable phase and most vulnerable to disrupted care-giving relationships. Intervening early is therefore imperative.

### Consequences of poor mother-infant interactions

Both short- and longer-term consequences have been reported for children of depressed and non-depressed mothers[19–22]. These can include poor social and cognitive outcomes from infancy to school age[6, 23–28], including poor psychological adaptation in adolescence[29], poor early school performance[30, 31], later anxiety[32], poorer self-regulatory capacities[33] and attachment insecurity[34] which in turn negatively affects subsequent interpersonal relationships[35], and has been linked with later behavioural problems[36]. As early as 3 months of age, infants of depressed mothers appear to generalise their depressed style of interaction to non-depressed adults[37].

Current evidence suggests that the mother-infant relationship is an important mediator between depression trajectories and child developmental outcomes (for example,[38]). In previous studies we demonstrated the critical importance of behavioural synchrony in the mother-infant interaction in the formation of attachment between mothers and their babies[39, 40]. More recently, we found that the quality (maternal responsiveness) of the early mother-infant relationship mediated poor child cognitive and behavioural outcomes at 4 years of age in a sample of women with PND[9]. Other evidence suggests ongoing difficulties to adolescence, particularly if depression is chronic[41, 42, 22].

### Treating mother-infant difficulties following postnatal depression

There is a critical need for brief interventions addressing mother-infant difficulties following PND, as treating maternal mood alone is not sufficient for improving mother-infant relationship difficulties[43]. A review of existing mother-infant interventions targeted at women with PND revealed that current studies of interventions were scarce, rarely used randomised controlled trial (RCT) methodology, are poorly evaluated, of long duration, and generally have not assessed infant outcomes[44–56]. In addition, many are not integrated with PND treatment of maternal mood symptoms. A brief mother-infant intervention that can change the developmental trajectory of infants of mothers with PND is of major public health significance and will potentially have important implications for preventing later emotional and behavioural disturbances in infants. In addition, this is most likely to improve outcomes if maternal depression is treated in order to facilitate the mother’s capacity to be emotionally available to her infant[7, 57]. This study aims to address the deficits in previous research: we use RCT methodology, an adequate sample size, an easily deliverable mother-infant intervention for mothers with PND that has been pilot tested, and assessment of both maternal and infant outcomes.

## Methods/Design

### Aims and objective

In a RCT, this study aims to evaluate the effectiveness of a targeted intervention (HUGS; Happiness, Understanding, Giving and Sharing) for enhancing mother-infant relationships.

The primary aim of the study is to determine whether women undergoing the combined PND treatment and HUGS programme will show greater improvement compared to a control group who received the PND treatment followed by an attention placebo playgroup (Playtime) in: 1) the observed quality of the mother-infant interaction; and 2) maternal reports of parenting stress, including feelings of attachment to their infant.

The secondary aim of the study is to determine whether infants undergoing the HUGS programme will show greater improvement compared to infants in the control condition in terms of: 1) difficult infant behaviour; and 2) early developmental milestones.

In addition, improvements in maternal mood are expected following the PND treatment and maternal mood is expected to continue to improve with the HUGS programme.

### Design

**Figure 1:**
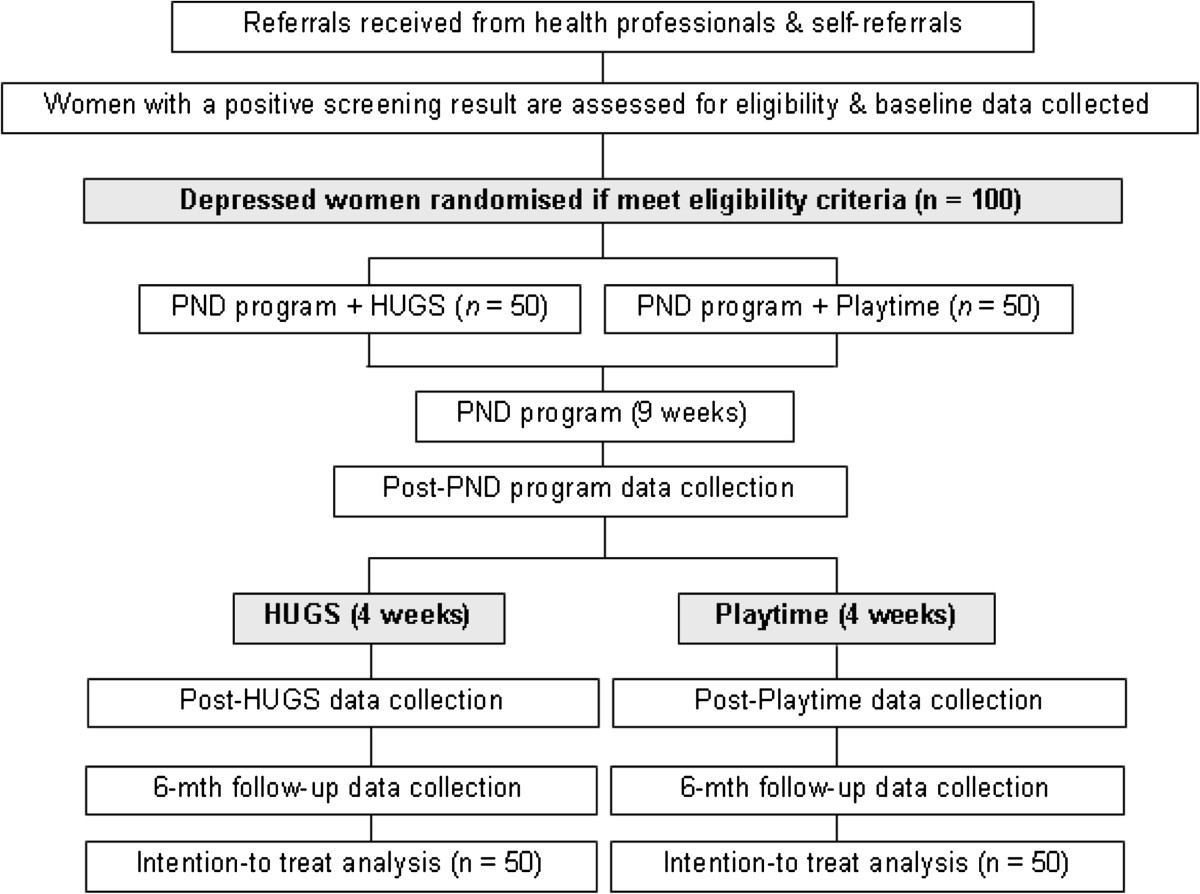
**Study flow diagram.** HUGS, Happiness Understanding Giving and Sharing; PND, postnatal depression.

The proposed research is a multi-site, parallel, two-group RCT involving 100 participants (n = 50 in each condition) and will be conducted in line with CONSORT standards ([58, 59]; http://www.consort-statement.org/). Figure 1 shows the design of the study.

### Procedure

Ethical approval has been obtained from Austin Health Human Research Ethics Committee (Project No. H2012/04745). Recruitment will be via referrals from health professionals including maternal and child health nurses and general practitioners, as well as self-referrals from women who have seen promotional materials for the study. They will be offered information sheets and consent forms where appropriate. After receiving informed consent, women will be screened with either the Edinburgh Postnatal Depression Scale[60] or the Whooley questions[61].

Women with a positive screening result (Edinburgh Postnatal Depression Scale ≥13 or an affirmative response to at least one of the two Whooley questions) will be assessed using the Structured Clinical Interview for the Diagnostic and Statistical Manual of Mental Disorders-IV-TR Axis I Disorders[62] to yield a diagnosis of current major or minor depressive disorder. Baseline questionnaires will be completed. Women who meet eligibility criteria will be randomly allocated to receive either HUGS or the attention placebo (Playtime) following the PND programme.

### Randomisation

Women are randomised prior to commencing the 9-week PND programme. A coded, double-blinded, variable-length permuted blocks randomised treatment allocation schedule produced by computer algorithm will be used. The allocation schedule will be stratified by site. It is not feasible to conceal the content of the intervention from those delivering the treatment. Participants will be blind to which condition is the intervention as both conditions will be presented as potentially beneficial.

### Inclusion and exclusion criteria

#### Inclusion criteria

Women with a clinical diagnosis of current major or minor depressive disorder as measured by the structured clinical interview, 18 years of age or older, and with an infant aged <12 months of age will be included in this study.

#### Exclusion criteria

Women fulfilling one or more of the following criteria will be excluded from participating in this study: currently receiving treatment for depression, significant difficulty with English, medium to high suicide risk, current self-harm, current substance abuse, current post-traumatic stress disorder, current manic/hypomanic episode or psychotic symptoms.

### Study outcome measures

The measures and time points of administration are shown in Table 1. The primary outcome measures are the Parenting Stress Index (PSI) and the Parent-child Early Relational Assessment (PCERA). All instruments to be used are established, well-validated, reliable and widely used, with well-described psychometric properties.

**Table 1:** **Summary and timing of measures**

	Baseline	Post-PND programme	Post-HUGS/ Playtime	6-month follow-up
**Maternal measures**				
Socio-demographics: marital status, education, occupation, ethnicity, income, number of children, depression history, child health.	✓			
Edinburgh Postnatal Depression Scale[60]: 10-items to screen for probable depression.	✓			
Beck Depression and Anxiety Inventories[63]: 21 items measuring depression/anxiety symptoms.	✓	✓	✓	✓
Structured Clinical Interview for the DSM-IV-TR Axis I Disorders[62]: this diagnostic interview was developed to assess major Axis I disorders using nomenclature derived from the DSM-IV. Mood disorders, post-traumatic stress disorder, substance use disorders, and psychotic screening modules will be included.	✓			
Programme evaluation: questions asking about participants’ satisfaction with treatment.		✓	✓	
Services accessed: questions asking about services participants have accessed since joining the study.		✓	✓	✓
**Mother-infant measures**				
Parenting Stress Index[64]: 101-items measuring dysfunctional parenting, 13 subscales including attachment and self-efficacy.	✓	✓	✓	✓
Parent-child Early Relational Assessment[65]: observational measure to assess the quality of the mother-child relationship. Videotaped sessions of unstructured play (10 minutes). Consists of mother, child and dyadic scales. Rated by a blinded trained observer. Used in over 200 studies.	✓	✓	✓	✓
Infant/Caregiver Behavioural Measure (Milgrom and Burn, unpublished data): videotaped sessions described above will also be scored by a blinded trained observer using this measure, which includes maternal, infant and interactional behaviours.	✓	✓	✓	✓
Postpartum Bonding Questionnaire[66]: the 12-item Impaired Bonding subscale will be used to detect mother-infant relationship problems.	✓	✓	✓	✓
**Infant measures**				
Short Temperament Scale for Infants/Toddlers[67, 68]: 30 items assessing temperament. Includes subscales reflecting difficult infant behaviours (irritability, cooperation/manageability). Normative data available for Australian children.	✓	✓	✓	✓
Ages & Stages Questionnaire[69]: assesses early developmental milestones in five domains (communication, gross motor and fine motor skills, problem-solving, and personal-social skills).	✓	✓	✓	✓
Ages & Stages Questionnaire Social-Emotional[70]: assesses infants’ social and emotional behaviour.	✓	✓	✓	✓

DSM, Diagnostic and Statistical Manual of Mental Disorders; HUGS, Happiness Understanding Giving and Sharing; PND, postnatal depression.

### Treatment

#### HUGS

Participants allocated to the HUGS treatment will receive the previously evaluated 9-week manualised PND group programme followed by four sessions of mother-infant HUGS group treatment as described by Milgrom and colleagues[71, 72]. The 9-week PND programme consists of nine cognitive behavioural therapy sessions and three evening couple sessions (12 sessions in total). The PND programme addresses maternal mood, behavioural activation, cognitive strategies, self-esteem, adaptation of relaxation training to focus on ways to “relax on the run”, the couple relationship, getting support, practical issues, problem-solving and self-care from a cognitive behavioural therapy framework. The HUGS sessions include psychoeducation and direct intervention in the mother-infant interaction. This builds on skills developed in the PND treatment programme, allowing a short duration “booster” to change the negative trajectory of mother-infant interactions. It has been successfully trialled in a feasibility study[10]. HUGS sessions include:

Session 1 Play and physical contact: play provides interactional opportunities. It allows assessment of interactional deficits and modelling of alternative responses.

Session 2 Observing and understanding baby’s signals: essential elements of a ‘good enough’ interaction are taught to parents using guided exercises to maximise small successes.

Session 3 Parental responses to infant cues: building on cognitive strategies learnt, distorted cognitions are challenged including separating past experiences from the reality of the infant. Infants can re-awaken powerful memories of earlier family relationships[73].

Session 4 Consolidating gains: reinforcing positive interactional behaviours and cognitions about the infant (booster session).

#### Control group

Participants allocated to the control group will receive the PND programme (described above). This is followed by four non-directive group meetings of mothers, infants and a facilitator in the same surroundings as HUGS but with no direct therapeutic work (acting as an attention placebo). This controls for possible effects of group membership, social networking and therapist contact. The “Playtime” playgroup has been developed to be consistent with what is currently provided in community playgroups and which mothers generally find supportive and includes four sessions providing an opportunity for informal discussion between mothers, some psychoeducation (for example, healthy eating) as well as time to play with babies.

## Data analysis

### Power calculations

The primary outcomes are post-treatment/follow-up scores on mother-infant relationship measures: the PSI and PCERA. Based on published parameter estimates on the PSI (baseline = 282.46, σ = 41.25) from Milgrom and colleagues[10], a between-group difference of (δ) 33 points would take scores into the normative range on the PSI (that is, ≤250). To detect a change of 33 points, the sample size per condition is *n* = 15.7 (14.25/33)^2^ = 24.53. For the PCERA we wish to detect a medium to large effect size (2/3 SD). This is within the range of effect sizes reported for a longer mother-infant intervention measured with the PCERA[45, 46]. This yields *n* = 15.7 (1/.66)^2^ = 36.04, which rounds to 40. Taking the highest estimate (PCERA) and allowing 10% attrition, the adjusted group is n* = *n*/(1-.10)^2^ = 49.38, which rounds to 50 depressed women in each condition.

### Analyses

Data will be screened to: (i) check for data entry errors; and (ii) test the assumptions underlying parametric procedures. For each outcome, *a priori* treatment comparisons will be conducted by fitting models controlling for baseline values, to yield direct comparisons of the effectiveness of the two conditions. Intention-to-treat principles following CONSORT guidelines will prevent systematic bias[59]. Baseline data will be secured prior to allocation, and missing values will be scrutinized to check for non-random distribution and imputed at the case level using gold-standard maximum likelihood methods (Multiple Imputation). Primary analyses will be executed twice: once using observed data, and once using multiple imputation methods given by Schafer[74], so that all 100 participants will be analysed in their allocated treatment condition. To assess how non-compliance affects results, the dose-response relationship between session attendance and level of clinical improvement will be explored. Primary data analyses will be conducted fully blind to treatment allocation (via coded treatment labels).

## Discussion

PND is prevalent and there is accumulating evidence that PND results in early dysfunctional relationships between mothers and infants with long-term consequences on infant brain development, cognitive functioning, emotional health and behaviour. For these reasons, there is a critical need for interventions addressing mother-infant difficulties following PND to explore whether later difficulties can be prevented. Mother-infant difficulties need to be addressed as soon as possible to prevent cumulative detrimental effects on child development[9, 10]. The HUGS intervention is innovative both in its brevity and conceptualisation. The vast majority of existing mother-infant interventions are intensive and/or long term. A brief, four-session intervention would be cost effective and could be rolled out to large numbers. Given the high prevalence of PND and that 70% of women with PND have relationship difficulties with their infants[9], a highly novel contribution of this study is the development of a brief, effective intervention that is easily taken up by primary care providers and added to existing treatment for PND.

Given the world-wide advocacy for universal screening for depression perinatally[75], there will be a sharp increase in the number of women identified and managed for their depressive condition. Best-practice pathways for women identified as being depressed perinatally need to be developed. A brief mother-infant intervention that can be added onto an existing treatment for PND would be an innovative way to provide a care pathway. In light of this, we developed the HUGS programme, a cost-effective, early intervention to bolster and protect the mother-infant relationship and prevent the intergenerational transmission of risk. The significance of early childhood experiences on adult health later in life is evident and, as such, the social and economic benefits of such an intervention are substantial.

## Trial status

At the time of manuscript submission, participant recruitment had not been completed. The trial is ongoing.

## Authors’ original submitted files for images

Below are the links to the authors’ original submitted files for images. Authors’ original file for figure 1

## Abbreviations

HUGS: Happiness Understanding Giving and Sharing
PCERA: Parent-child Early Relational Assessment
PND: postnatal depression
PSI: Parenting Stress Index
RCT: randomised controlled trial.
