# Supplementary figures and images for: Early intervention to protect the mother-infant relationship following postnatal depression: study protocol for a randomised controlled trial
Source: Trials. 2014 Oct 3;15:385. doi: 10.1186/1745-6215-15-385 (PMC4195961; doi:10.1186/1745-6215-15-385)

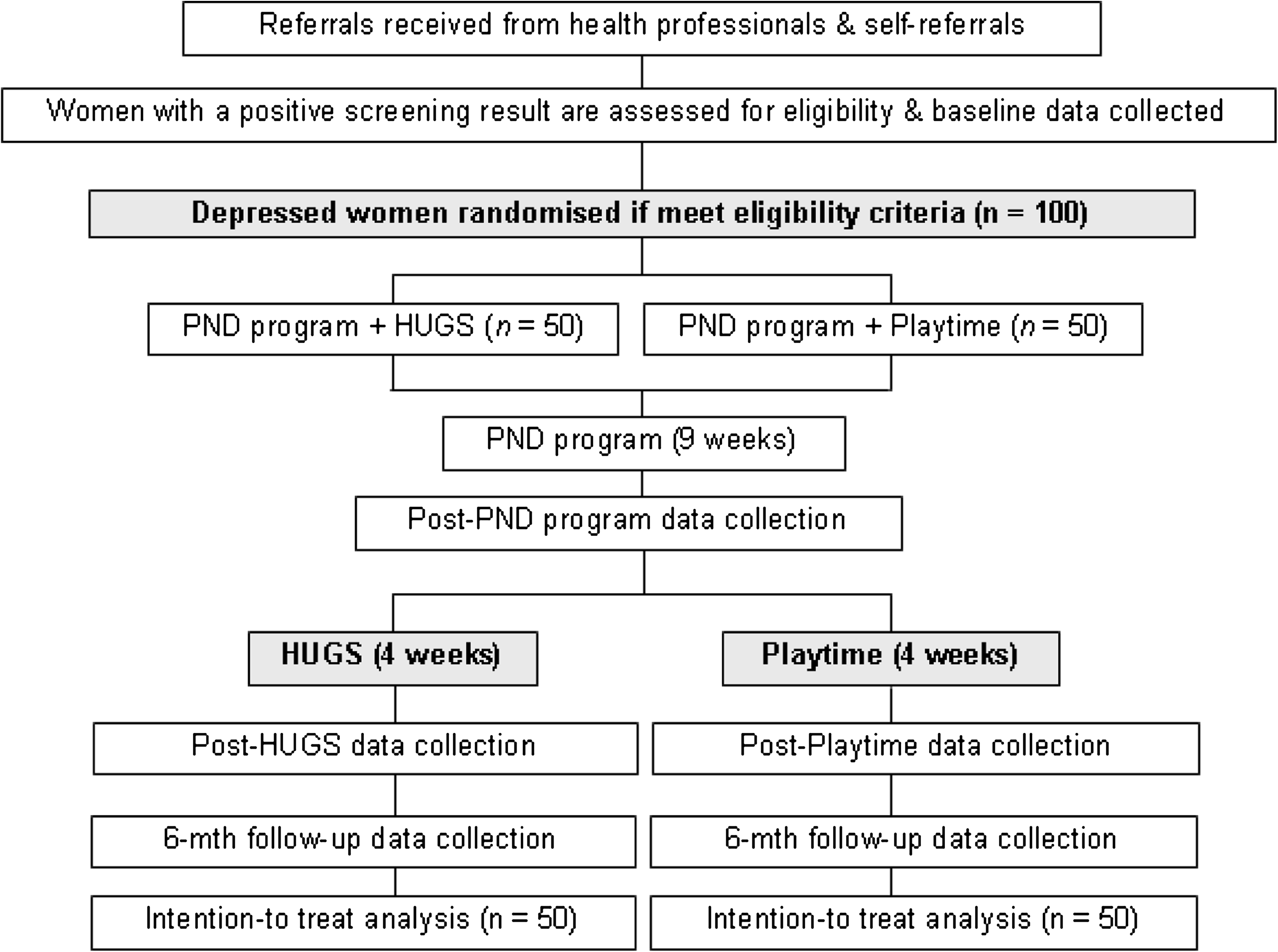

Supplement: Supplementary file 1 — Authors’ original file for figure 1 [file 13063_2014_2253_MOESM1_ESM.tif]
